# Book Reviews

**DOI:** 10.1038/sj.bjc.6690311

**Published:** 1999-04


*British Journal of Cancer* (1999) **79**(11/12), 1944–1945
© 1999 Cancer Research Campaign
Article no. bjoc.1998.0311

### Book Reviews

### The Biological Basis of Cancer

RG McKinnell, RE Parchment, AO Perantoni and GB Pierce
Cambridge, USA, Cambridge University Press, 1998, pp 378, £22.95, ISBN 0521 596955

This paperback is based on an undergraduate course of the same name at the University of Minnesota, using cancer as a vehicle for teaching molecular and cellular biology. The book opens with a series of anecdotes illustrating a variety of clinical problems in cancer, which form a background for the opening chapters on cancer pathology, metastasis and carcinogenesis. Each chapter is clearly subdivided into sections with headings such as 'Are there genes that control metastasis; Is metastasis limited to malignant cells?, and Why study metastasis?'

Chapter 1 defines terminology, compares benign and malignant cells, and describes the mechanism of deleterious effects of cancer. Initiation, development and progression of malignancy has been dealt with. A tabular presentation of differences between benign and malignant lesions would have been useful. Chapter 2 discusses cellular, enzymatic and genetic control mechanisms of cancer and steps in the metastatic cascade. Chapter 3 deals with carcinogenesis: carcinogens, modulation of carcinogenesis, tumour promotion and tumour progression. A passing remark on the Federal regulations for decreasing exposure to carcinogens is also included. Taking into account the people for whom the book is basically intended, the large abundance of complicated chemical structures of carcinogens could have been easily avoided. Chapter 4 on cancer genetics discusses chromosomes and cancer, hereditary cancers, and familial cancer syndromes. A brief write-up on chromosomal and gene structure would have been a useful addition. Chapter 5 introduces the concept of oncogenes. It discusses proto-oncogenes and dysfunction in signal transduction and cell cycle regulation or differentiation. Interaction with carcinogen and oncogene activation has been dealt with. The section on suppressor genes discusses Rb, p53 and other suppressors. Chapter 6, which deals with 'Cancer in non-human organisms', has been included with a justification that, firstly, scrutiny of lower organisms provides information directly useful in understanding human biology and, secondly, phyletic approach to cancer relates to the finding of organisms that are relatively or absolutely resistant to cancer and, finally, experimental organisms permit novel and unusual approaches to this aspect of cancer research. Chapter 7 discusses the epidemiology of selected human cancers and issues such as occupational cancers, AIDS-related cancers, diet, nutrition and cancer, exercise as it relates to cancer, and tobacco as a lifestyle hazard. Chapter 8 is a discussion on the biological basis of the modalities of cancer treatment. The contents under the heading 'Cytoreduction theory and cancer cure' discuss absolute versus fractional cytoreduction, cancer cure success and failure of multimodality therapy and complicating factors that decrease log-cell kill. Chapter 9 describes the therapeutic approaches that aim to alter the biology of malignancy so that its growth can be controlled. The issues of drug targeting using markers, trophic factors in tissue renewal and therapy and differentiation therapy which describes treatments that seek to alter the balance between proliferation and differentiation in a tumour and reset homoeostasis have been addressed.

Finally, the appendix gives a description of selected tumours. A glossary of cancer-related terms has been included.

The text of the book is presented in an easily comprehensible style. Although the style of presentation is fine, it would have been very useful to have terms and salient features of the text in italics, in bold or within parentheses which could facilitate easy reference. It would have been very useful had there been included more line and schematic diagrams and flow charts with regard to the different oncogene and tumour-suppressor gene pathways, cell cycle and tumour control pathways, enzyme and chemical activity in metastatic pathway. This could lead to a better understanding of the narrative text. An elaborate discussion on cell cycle, the phase and the relevance to tumour growth and treatment would have been a useful part of the book. A section or chapter on the various important cancer biology laboratory techniques would have been most welcome. A section or discussion on tumour immunology and the relevance to tumour growth and treatment would have been useful.

This book can be useful to undergraduate medical students and biology students for the understanding of basic concepts of cancer biology. It could also be a useful reference book for clinicians and cancer biology researches at the beginning of the career. It could be a useful reference for students preparing for part I examination in clinical oncology or for students pursuing a course in medical oncology, but not as a main text.

*John Yarnold*
*Department of Radiotherapy,*
*Royal Marsden Hospital,*
*Downs Road,*
*Sutton,*
*Surrey SM2 5TT*

### Tumour Angiogenesis

Edited by R Bicknell, CE Lewis and N Ferrara.
Oxford University Press: Oxford, 1997, £85

Angiogenesis, the development of new vessels from pre-existing vessels, is of key importance in nearly all normal and pathological growth processes. The eminent role of new vessel formation in general was already recognized in the 18th century. Pathologists such as Virchow and Thiersch stressed the significance and morphological differences of the tumour-inherent vasculature more than 130 years ago. However, more than a century of quiescence passed before a revival of the scientific interest in angiogenesis occurred. Today's wide interest in this topic is, at least in part, the result of the enormous socio-economic impact of cancer (and other angiogenic diseases) as well as the result of the outcome of conventional therapeutic regimens.

Visionary concepts of scientists such as Judah Folkman, who has proved that solid tumours are absolutely dependent on angiogenesis for growth beyond 1–2 mm, have stimulated the scientific community to focus on nearly all aspects of the induction, regulation and manipulation of angiogenesis. The outcome of these efforts is a dramatic increase in our knowledge of normal and pathological angiogenesis from the molecular biological level to preclinical and clinical treatment studies.

For instance, the majority of clinicopathological studies confirm a strict association between angiogenic activity of a primary tumour and its potential to metastasize. Additionally, several studies have shown, in a variety of tumours including breast and ovarian cancer, that the intratumoral microvascular density is one of the most powerful prognostic indicators. Recent experimental strategies focus on the vascular and/or angiogenic attack of tumours, taking advantage of the avalanche effect of depriving tumour cells of their blood supply rather than directing monoclonal antibodies against the tumour cells themselves.

However, it should be kept in mind that up until now only very few of the more than 200 identified anti-angiogenic compounds have shown anti-tumour effect. On the other hand, these results, as well as the idea of anti-angiogenic gene therapy experiments involving the blockage of VEGF receptors are very promising.

*Tumour Angiogenesis* attempts to comprehensively cover all areas of this rapidly expanding research field. The editors have succeeded in bringing together numerous leading experts in the field giving state of the art reviews of the individual topics in 27 chapters representing the knowledge of 1997. Nearly all contributions can be read independently, making it easier both for beginners and experts in the field. More than 2800 references prove that the authors have put a lot of effort into providing a more or less complete and comprehensive bibliography.

The development of the embryonic vascular system is also addressed, together with a profound presentation and discussion of the relevant in vivo models of angiogenesis. Physiological basics of the tumour vascular system and the impact of microregional blood flow elucidate the role of the vascularity and provide the basis for a better understanding of the difficulties encountered in all therapeutic modalities including angiogenic and vascular attack.

The role of oncogene activation and tumour suppressor gene loss are also highlighted against the background of recent findings that the same genes are also responsible for the induction of neovascularization.

Special emphasis is given to the complex field of polypeptide factors, cytokines, their soluble receptors and antagonists as well as the proteolytic enzymes regulating their release. Even though we do not yet have a widely accepted scheme of the orchestration and significance of the individual factors, these chapters contribute to a far better understanding of what is essential for new vessel growth. This is also true, e.g. for the role of intracellular signalling agents like nitric oxide, where we also face a dramatic expansion in knowledge.

Perhaps the most interesting and promising chapters deal with the role and possibilities of the VEGF system. The FGF family is still recognized as an important player in tumour angiogenesis, especially when tumours increase and invade by degrading the extracellular matrix. Recent findings, however, including the knockout of the VEGF gene, provided direct evidence for an irreplaceable key role of VEGF in the normal development of the vascular system.

The readability and understanding of these complex chapters is greatly enhanced by the contributors' efforts to highlight the most relevant findings in close context with other aspects of tumour angiogenesis.

In summary, *Tumour Angiogenesis* is a very stimulating, reliable and comprehensive book of great value both for experts and junior researchers. It is always somewhat dangerous to issue a book in a rapidly growing field because of the imminent expiry date. We believe, however, that this handbook will be of great value even in a couple of years.

*MA Konerding[1] and P Vaupel[2]*
*[1]Institute of Anatomy,*
*[2]Institute of Physiology & Pathophysiology*
*University of Mainz*
*Duesbergweg 6*
*D-55099 Mainz, Germany*

*© Cancer Research Campaign 1999*

